# Infant Viability in Severe Preeclampsia: Management Strategies and the Potential Role of Calprotectin—A Narrative Review

**DOI:** 10.3390/children12101410

**Published:** 2025-10-18

**Authors:** Oala Ioan Emilian, Adrian Apostol, Viviana Mihaela Ivan, Lucian Pușcașiu

**Affiliations:** 1 Doctoral School, Faculty of Medicine, “George Emil Palade” University of Medicine, Pharmacy, Sciences and Technology, 540142 Targu Mures, Romania; lucian.puscasiu@umfst.ro; 2 Department of Cardiology, “Victor Babes” University of Medicine and Pharmacy, 2 Eftimie Murgu Sq., 300041 Timisoara, Romania; adrian.apostol@umft.ro (A.A.); ivan.viviana@umft.ro (V.M.I.)

**Keywords:** preeclampsia, intrauterine growth restriction, management, calprotectin, infant viability

## Abstract

Preeclampsia (PE) is a vascular-related pregnancy disorder characterized by high blood pressure and proteinuria after 20 weeks’ gestation. Defective placentation, together with endothelial dysfunction, has a crucial role in the development of PE. Current evidence suggests that calprotectin is a potential marker for screening, even if it is not yet a standard diagnostic tool. The aim of our study is to the review monitoring methods for severe preeclampsia, which endangers neonatal viability. Starting from here, we look for ways to safely prolong pregnancy and also evaluate calprotectin as a potential biomarker of this pathology. Current issues and future perspectives are analyzed. As a solution, multidisciplinary management should be offered in tertiary-level units by maternal–fetal medicine specialists and neonatology units to increase fetal/neonatal viability. Based on the severity of preeclampsia and intrauterine growth restriction, cardiotocography and Doppler ultrasound monitoring should be scheduled. Delivery is also taken into consideration based on gestational age and maternal condition. Placental histological findings appear to be crucial in understanding this disease. The elevated calprotectin levels in preeclampsia suggest underlying inflammatory processes in the mother, which potentially contribute to the development of the condition; however, more research is needed to clarify calprotectin’s role. Conclusion: Early-stage preeclampsia remains a significant risk to maternal and neonatal health, with significant impacts on neonatal viability. Further elucidation of a role for calprotectin in the development of preeclampsia and its relevance for fetal viability are necessary. Calprotectin could be a potential biomarker in preeclampsia, as an important inflammation marker. But, so far, calprotectin has failed to prove its role as a marker of fetal viability, and thus, more studies are needed.

## 1. Introduction

Preeclampsia is a pregnancy condition characterized by high blood pressure and proteinuria after 20 weeks of gestation, and is associated with significant morbidity and mortality as one of the leading causes of perinatal and maternal mortality worldwide [1]. It can be accompanied by acute kidney injury, liver dysfunction, and fetal intrauterine growth restriction in the second half of pregnancy. Preeclampsia (PE) affects 5% of pregnancies worldwide [1,2,3,4] and is the most common cause of premature delivery [2,4]. Currently, there is no treatment for preeclampsia except delivery of the baby and placenta.

PE is frequently accompanied by intrauterine growth restriction (IUGR) [5,6]. Risk factors for IUGR are maternal conditions such as chronic hypertension or diabetes, maternal age over 40 years, smoking, abuse of alcohol and drugs, severe malnutrition, preeclampsia, placental malperfusion, or fetal abnormalities. A woman who has had a baby with IUGR in the past is at risk of having another baby with the same condition. IUGR can lead to adverse perinatal outcomes, such as a low birth weight, preterm birth, or other complications [6,7].

Preeclampsia (PE) is a vascular-related pregnancy disorder with unclear etiology [2,8,9]. A normal pregnancy is characterized by a series of complex processes, and any alteration to this chronological progression can have significant effects on the mother and the fetus [10]. Preeclampsia is associated with dysregulated remodeling of the spiral arteries within the placental bed, associated with oxidative stress [2,8,10]. This defective placentation, together with endothelial dysfunction, plays a crucial role in the development of PE [10]. This condition leads to an increased risk of cardiovascular diseases (CVDs) later in life [8].

Placental malperfusion leads to placental ischemia/hypoxia with the release of anti-angiogenic factors, such as soluble FMS-like tyrosine kinase 1 (sFlt1) and soluble endoglin (sEng) [8,10]. Soluble FMS-like tyrosine kinase 1 (sFlt1) is the main anti-angiogenic factor highly relevant as biomarker for PE with a negative effect on the endothelium. It is used in the first trimester screening, along with pregnancy-associated placental protein-A (PAPP-A) and placental growth factor (PlGF), which decrease in PE [10,11].

Another biomarker sought in preeclampsia pathophysiology is calprotectin. Calprotectin is a complex of two calcium-binding proteins S100A8 and S100A9. S100 is part of a family of low-molecular-weight calcium-binding proteins (9–14 kDa) required for inflammation, and is also involved in cellular homeostasis, such as calcium storage and transport [12,13]. It can be found in the cytosol of neutrophils which are activated in preeclampsia, potentially contributing to its pathophysiology [14]. S100 isoforms have important roles in the immune system as alarmins, pro-inflammation stimulators with an antimicrobial role, and metal scavengers. In addition, the decidua has been shown to have a role in severe PE [13].

The aim of our study is to review monitoring methods for severe preeclampsia, which endangers neonatal viability. Starting from here, we look for ways to safely prolong pregnancy, and also evaluate calprotectin as a potential biomarker of this pathology.

## 2. Materials and Methods

**Figure 1:**
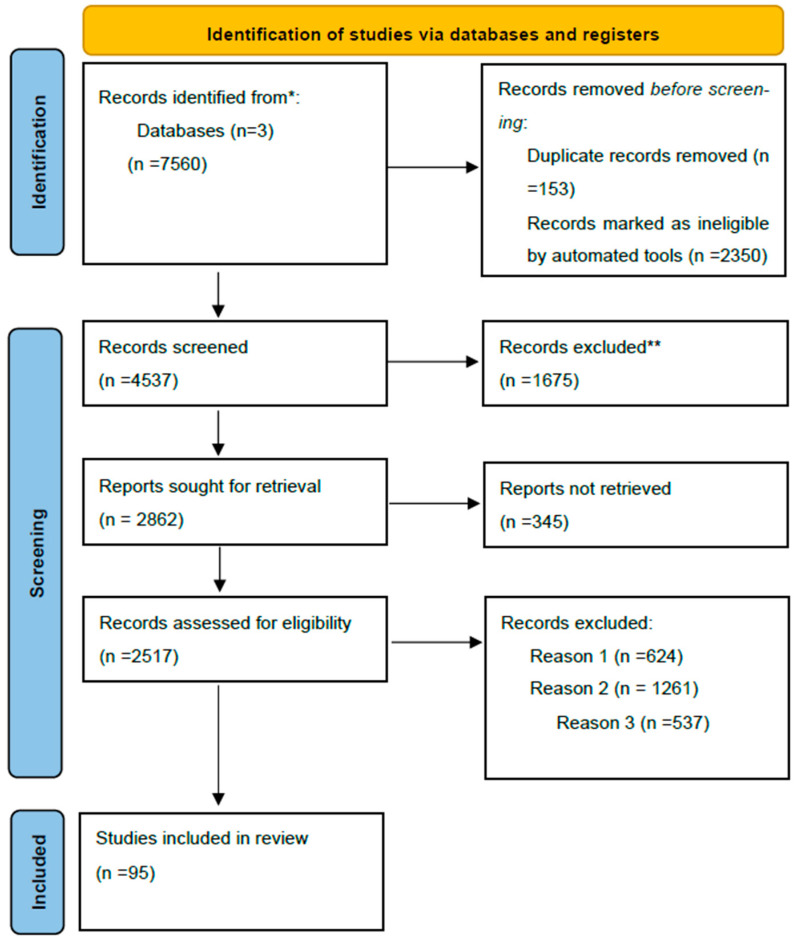
Flow diagram of the article selection method. * The number of records identified in Google Scholar, PubMed and Cochrane databases. ** Records excluded by a human.

Our search strategy was conducted in 2025, focusing on publications from the past three decades using the following specific keywords: “preeclampsia”, “infant viability”, “biomarkers”, “placenta”, and “calprotectin”. A thorough literature review spanning a period of the last three decades was conducted on electronic databases such as Google Scholar, PubMed, and Cochrane. The search generated 7560 articles. After carefully screening abstracts, removing duplicates and conducting full-text reviews, we retained 95 full-text articles written in English with methods clearly defined and exhibiting appropriate study design, safely prolonging gestation, increasing infant viability as primary outcomes. Also, calprotectin was evaluated as a potential biomarker. Editorials, literature reports, and studies not aligned with the objectives of this review were excluded. Two independent authors selected full-text articles and adopted a narrative approach given the impracticality of data pooling, the heterogeneity of the study design, and outcomes reported by the researchers. They used SANRA, a quality scale for the assessment of narrative review articles to rate the justification of importance of the articles included, and scientific reasoning. The article selection process is visualized in the following flow diagram (Figure 1).

Reason 1: Records excluded due to publication in a language other than English.

Reason 2: Records excluded by a human reviewer due to inaccurate or inappropriate titles.

Reason 3: Records excluded based on the study’s research design.

## 3. Short Definition and Monitoring of Preeclampsia

Preeclampsia is a condition that develops after 20 weeks of gestation, characterized by hypertension and proteinuria in normotensive patients before pregnancy. Other signs or symptoms in some women without proteinuria may occur. Reliance on observations of preeclampsia symptoms could be problematic in clinical practice. Hypertension during pregnancy is defined as a systolic blood pressure of 140 mm Hg or more or a diastolic blood pressure of 90 mm Hg or more, or both, measured at least on two occasions 4 h apart. Severe preeclampsia is diagnosed when women have severe blood pressure values (TAS > 160 mm Hg or higher or TAD > 110 mm Hg or higher), which increases the risk of morbidity and mortality [1]. In addition to the classic PE criteria, which are HTA and proteinuria, other features must be taken into account:

- Thrombocytopenia (Tr,100,000 3 109/L);
- Impaired liver function;
- Severe epigastric pain;
- Renal insufficiency in the absence of other renal disease;
- Pulmonary edema;
- Headaches;
- Visual symptoms [1].

In this case, fetuses are at increased risk of high morbidity, mostly due to preterm delivery, at the limit of viability. Women with PE are at risk of cardiovascular disease later in life. Several studies have linked preeclampsia with an increased risk of myocardial infarction, hypertension, congestive heart failure, stroke, and cardiovascular mortality in subsequent years compared with women unaffected by preeclampsia [9,10,15,16].

Unfortunately, there is no effective and definitive treatment for preeclampsia yet, except delivery of the baby and placenta. Morbidity and mortality also depend on the disease severity and gestational age. Developing a form of therapy that could safely prolong the duration of pregnancy is of enormous value in the area of preeclampsia management, especially in women with early-onset severe preeclampsia [16].

The definitions of the International Society for Ultrasound in Obstetrics and Gynecology (ISUOG) and the Society for Maternal–Fetal Medicine (SMFM) also take into account placental histopathological findings associated with placental insufficiency that leads to adverse neonatal outcomes [7]. The fetal growth restriction outlined in the ISUOG and SMFM’s PE definitions has a limited discriminatory capacity for placental histopathological findings associated with adverse neonatal outcomes and infant viability [7].

The evaluation of IUGR is a key objective of prenatal management. It depends on several factors, such as uteroplacental perfusion; maternal disease, nutrition, smoking, and drug use; and altitude. Pathological conditions, including infection, or some genetic conditions can also influence fetal growth. However, uteroplacental insufficiency represents the most frequent cause of IUGR in a normal fetus. This condition influences fetal/infant viability, with long-term adverse outcomes. IUGR is most frequently associated with prematurity, but is sometimes accompanied by worse neurodevelopmental outcomes. Other conditions developed by infants with IUGR include increased risk of hypertension in adulthood, metabolic syndrome, insulin resistance, coronary heart disease, and stroke [17]. Women are at risk of developing atherosclerotic cardiovascular disease later in life, with the underlying mechanisms of this unclear [18].

### Monitoring the Onset of PE and IUGR

It is necessary to evaluate fetuses that deviate from their individual growth trajectory using biometric measurements [17]. The application of Doppler velocimetry is also mandatory in fetal growth management to identify the uteroplacental function in PE. In this regard, Doppler velocimetry through the uterine and umbilical arteries is necessary. Uteroplacental insufficiency is mediated through spiral artery defective remodeling, which reflects impaired trophoblastic invasion, leading to high-resistance circulation. On the fetal side, Doppler velocimetry should be used to evaluate the middle cerebral artery and ductus venosus, which would allow for the observation of the fetal cardiovascular adaptation from hypoxia to acidemia [17]. A high uterine artery mean pulsatility index (PI) (above the 95th percentile) is linked with placental insufficiency [9,17].

Cardiotocography allows us to monitor the fetal heart activity, which could be altered in the context of IUGR. Reactive cardiotocography (CTG) excludes fetal hypoxemia. A reduced fetal heart rate variation accompanies severe hypoxemia or hypoxia [17].

Some placental proteins, such as pregnancy-associated plasma protein-A (PAPPA) and soluble fms-like tyrosine kinase-1 (sFlt-1), as well as the placental growth factor (PlGF) ratio and elevated levels of vascular endothelial growth factor receptor-1 (VEGFR-1), are biomarkers of placental function in the first trimester with predictive abilities. However, their value is limited [9,17,19]. It has been already demonstrated that calprotectin (S100A8/S100A9) is released in ischemia/reperfusion injury [20,21,22,23]. The hypothesis that S100A8/A9 oxidation releases S100A9 under mild oxidative conditions and is involved in PE pathophysiology remains controversial and needs further clarification [20]. Calprotectin may be future biomarkers of endothelial damage during pregnancy [24].

Placental histopathological findings are also crucial in identifying poor trophoblast implantation and spiral artery abnormalities, which lead to maternal vascular malperfusion [17,19].

There are two main phenotypes of PE. These two types not only differ significantly with regard to the gestational age at onset and prediction in first-trimester ultrasound, but also in placental histopathological and Doppler ultrasound findings; various maternal diseases are involved. Early-onset PE has the highest severity, with poorer perinatal outcomes. The optimal gestational age at diagnosis would be 32 weeks, although this is not always easy as a single biometric measurement is not enough to evaluate fetal growth. Monitoring the progress of early- or late-onset PE is crucial for PE management [17]. The definition of IUGR was established by the International Society for Ultrasound in Obstetrics and Gynecology (ISUOG) and the Society for Maternal–Fetal Medicine (SMFM) to evaluate fetal outcomes [7].

Multidisciplinary management should be offered in tertiary-level units by maternal–fetal medicine specialists and neonatology units to increase fetal/neonatal viability. CTG and Doppler ultrasound monitoring should also be scheduled based on the severity of IUGR. In addition, delivery should be taken into consideration based on gestational age and maternal conditions, such as persistent symptoms of severe PE, eclampsia, HELLP syndrome (hemolysis, elevated liver enzymes, low platelets), pulmonary edema, disseminated intravascular coagulation, renal insufficiency, and obstetric emergency. Management of selected patients can improve neonatal outcomes, reducing the number of neonatal intensive care admissions (NICAs); for example, personalized management should be offered at 24–26 weeks to increase perinatal viability [9,17,24].

## 4. Placental Malperfusion and Related Biomarkers

Placental histological findings have been found to be crucial in understanding this disease. The placenta has a pivotal role in facilitating optimal intrauterine fetal development. Even so, the placenta and the placental function remain enigmatic [19] due to the ethical constraints of studying early placental development. Implantation success depends on both embryo quality and a normal endometrium state, and the disease begins with abnormal remodeling of uterine spiral arteries during placentation [9].

Four major patterns of placental injury have been described by the Amsterdam consensus: maternal vascular malperfusion (MVM), fetal vascular malperfusion (FVM), Acute Chorioamnionitis (ACA), and Villitis of Unknown Etiology (VUE). The aim of these markers is to improve the dialog between clinicians and pathologists. MVM can be described as a placental insufficiency characterized by decidual arteriopathy, accelerated villous maturation and increased fibrin deposition, while FVM consists of fetal blood vessel disruption within the placenta, leading to ischemia and thrombosis, including umbilical cord thrombosis and fetal vasculopathy [25].

Placental insufficiency can lead to adverse neonatal outcomes and is correlated with placental histopathological findings [7]. Its dysfunction underlies a series of pathologies, including preeclampsia and IUGR [26]. Placental lesions are associated with maternal placental malperfusion and are correlated with a low Apgar score, NICA, respiratory distress syndrome, and perinatal death [7]. Decidual samples have been obtained from women with PE, and acute atherosis was detected in placental bed decidua. Acute atherosis is defined as the presence of at least two intramural vacuolated cells on CD68 staining, demonstrating high reproducibility (inter- and intra-observer agreement). These findings were found to be correlated with a lower gestational age and lower birth weight percentile at delivery, with the authors mentioning that the studied women were not known to exhibit conventional cardiovascular disease risk factors [18].

Preeclampsia is characterized by deficient trophoblast invasion and maternal endothelial dysfunction, with maternal leukocyte activation and possible implication of calprotectin [3,12]. Genetic mechanisms and risk factors are involved in its etiology [4], while the fibrinolytic system also plays an important role in its evolution [27]. Mitochondria are the most important cellular producer of reactive oxygen species, and are a pathogenic mediator of oxidative stress in preeclampsia, along with the innate immune system [2]. Additionally, a significant increase in the production of neutrophil activation markers, such as calprotectin and interleukin-8, has been observed in women with preeclampsia, while sFlt-1, an anti-angiogenic factor, was decreased [2]. Circulating levels of anti-angiogenic factors may be used as a biomarker for the early diagnosis of preeclampsia (PE) [11].

Placental apoptosis is increased in PE and intrauterine growth restriction (IUGR). The causes of this are unknown [28]. Apoptosis, i.e., programmed cell death, is an important phenomenon in the development of human tissues, including the placenta. Trophoblast apoptosis is a normal event in placental aging, but elevated levels of placental apoptosis have been observed in intrauterine growth restriction (IUGR) and preeclampsia (PE) [28].

Angiogenesis-related factors, such as sFlt-1 (soluble fms-like tyrosine kinase 1), PlGF (placental growth factor), and vascular endothelial growth factor (VEGF), have a crucial role in placental dysfunction. Altered levels of these biomarkers can help us to predict maternal and fetal outcomes [19,26].

Analysis of angiogenic biomarkers with or without uterine Doppler ultrasound substantially improves the sensitivity and specificity of predictions of adverse outcomes. Some researchers have proposed extending the definition of preeclampsia in the future to include altered angiogenic factors (sFlt-1/PlGF ratio or PlGF alone), which indicate placental dysfunction [26].

These biomarkers can be correlated with clinical characteristics and ultrasound findings to improve the prediction of PE in the first trimester [26]. Endothelial function was assessed in Kvehaugen et al.’s study, in which a significantly reduced endothelial function was identified in both mothers and children after pregnancy following PE associated with IUGR compared with mothers and children without this condition [8]. Levels of maternal soluble fms-like tyrosine kinase 1 were elevated postpartum in the preeclampsia group compared with controls. This reduced function of the endothelium in both mothers and children was observed 5 to 8 years after PE pregnancies [8].

Genetic biomarkers have also been sought, and circular RNA might be involved in early-onset preeclampsia. Trophoblastic cells are invading the spiral arteries because of this aberrant molecule. The dysregulation of chromosome 19 microRNA has an impact on angiogenesis in the placenta, resulting in PE. Other gene dysregulation involved in IUGR and PE are CD40L, TNFRSF8, and IL1R2, which modulate inflammatory response [29]. Other gene variants with an important role in PE are genes responsible for DNA repair, inflammation-related genes, or apoptosis-related genes. Fetal cell debris, specifically trophoblast debris that enters maternal circulation, promotes an intense inflammatory response that plays an important role in the predisposition to develop PE. Several immune-related genes have been evaluated; CTLA4, CD28, IL1R1, and TGFB1 single-nucleotide polymorphisms (SNPs) as well as ICOS SNPs seem to have a role in PE [30]. Genetic polymorphisms involved in oxidative stress in endometriosis lead to PE in the case of pregnancy [31].

## 5. Perinatal Outcomes and Infant Viability

In a study by Bombrys et al. (2008), 46 patients with severe PE at less than 27 weeks were studied, and it was concluded that perinatal outcomes and viability are dependent on the gestational age both at onset of PE and at delivery. Maternal complications were also evaluated, and it was found that at less than 24 weeks, in the case of a high maternal morbidity combined with extremely low perinatal survival, termination of the pregnancy should be offered. Extensive counseling is necessary for the patients [32].

In a study by Witlin et al. (2000) of 195 pregnancies for which the baby was delivered between 24 and 33 weeks gestation, it was found that respiratory distress syndrome was inversely related to gestational age at delivery and directly related to cesarean section, while survival was directly related to birth weight and corticoid use exhibited no correlation with neonatal morbidity [33].

There is no effective or definitive treatment for preeclampsia, except delivery of the baby and placenta. Infant viability depends on the disease severity and gestational age [16]. The significant role of angiogenic factors must be taken into account in the etiology of preeclampsia. This could lead to their future use in diagnosis and risk assessments of the disease. These findings can also open a new avenue in the field of treatment of this serious pregnancy complication, or be used to develop a therapy that could safely prolong the duration of pregnancy, especially in women with early-onset severe preeclampsia. This would be invaluable in lowering perinatal complications [16].

Impaired fetal growth associated with PE is correlated with an increased risk of perinatal mortality and morbidity, leading to long-term adverse infant outcomes. This condition is also associated with prematurity and poorer neurodevelopmental outcomes. These infants are also at risk of developing hypertension, coronary heart disease, metabolic syndrome, and stroke in adulthood [17]. The perinatal outcomes of both mothers and fetuses were stratified by Shear et al. (2005) according to gestational age and the severity of IUGR. The authors strongly recommended expectant management in fetuses at <30 weeks of gestation, irrespective of fetal growth restriction, to improve infant viability [34].

## 6. Calprotectin as a Potential Biomarker in PE and Its Relationship with Neonatal Viability

Calprotectin is an inflammation marker found in the cytosol of leukocytes, and plays a role in various physiological processes, such as defense against bacterial infections. It may also be elevated in the plasma of women with preeclampsia, suggesting a maternal inflammatory response. Preeclampsia is associated with maternal leukocyte activation [35]. The role of calprotectin has not previously been explored in PE. Braekke et al. (2005) found elevated levels of calprotectin in maternal circulation in preeclamptic pregnancies compared with normal pregnancies in their study, without evidence of inflammation in fetal circulation. This indicates that inflammation is primarily a maternal phenomenon [36].

Some researchers suggest that calprotectin might contribute to the pathogenesis of PE [37]. More research is needed to understand its role in diagnosis and as a prognostic marker [38].

The calprotectin level is elevated in inflammation, for example, during COVID-19 infection in pregnant patients, and Michaud et al. (2022) found that pregnant patients with COVID-19 infections are twice as likely to develop preeclampsia compared with non-COVID-19 pregnant women [39].

Holthe et al., in their study (2005) of 52 women (20 with PE, 20 normotensive pregnancies, and 12 nonpregnant women), discovered significantly elevated plasma calprotectin levels in preeclamptic patients compared with matched normotensive pregnancies. This finding supports the notion that leukocytes are activated in preeclampsia. Further evaluation of the role of calprotectin in the development of PE is required [12]. Evidence supports that calcium is a calprotectin sensor [40], and it has also been demonstrated that placental insufficiency in early pregnancy can lead to elevated levels of plasma calprotectin, in turn leading to PE [41,42,43].

In a review covering seven studies of 245 women with preeclampsia and 194 healthy controls, it was found that serum calprotectin levels were significantly elevated among preeclamptic patients (*p* < 0.05). However, to date, there is no specific cut-off value to help screen women for preeclampsia [14].

Calprotectin levels were evaluated in the amniotic fluid of 79 women with preterm labor to assess inflammation and infection, which could influence perinatal viability [41,44]. Amniotic fluid was collected and analyzed, and calprotectin and IL-6 levels were measured. In addition, microbial invasion was evaluated using positive PCR or positive culture. Calprotectin concentrations in amniotic fluid were significantly higher in the intra-amniotic infection group compared with the other groups [44].

Godtfredsen’s 2022 study on 117 women with PE demonstrates that PE is associated with coagulopathy and increased fibrinolysis, which leads to thrombosis [27].

It is known that calprotectin is associated with neutrophil extracellular traps (NETs). These NETs were elevated in the decidua and placenta of women with PE and HIV infection [45], showing that NETs are released by neutrophils, maybe as a last resort, to control microbial infections [46]. In Moodley et al.’s (2020) study, a new marker to localize NETs was found: histone H2A expression. A significant decline in H2A immuno-expression was observed in preeclamptic women infected with HIV compared with non-infected women. Elevated placental NETs have been observed in both PE and HIV-infected patients [45], and a pathogenic role of calprotectin and NETs was observed in PE by Kaplan et al. in their study [47].

Neutrophil extracellular traps (NETs) take part in the neutrophil pathogen-killing mechanism, a type of programmed neutrophil death, and consist of chromatin and histones along with serine proteases and myeloperoxidase. NETs are induced by a variety of infectious and non-infectious *stimuli*. Neutrophil dysregulation has been demonstrated in the pathogenesis of vascular diseases. NETs are an important source of autoantigens in autoimmune diseases, including systemic lupus erythematosus (SLE), rheumatoid arthritis (RA), and thrombosis, as well as in the pathogenesis of cancer [48,49,50].

There is a gap in the understanding of the correlation between antiphospholipid syndrome (APS) and preeclampsia (PE). Antiphospholipid antibodies (aPLs) act in two different ways: they induce a pro-coagulant state, leading to thrombosis, and directly impact trophoblastic cells, influencing spiral artery remodeling [9].

Systemic lupus erythematosus (SLE) and antiphospholipid syndrome (APS) are two autoimmune diseases that can occur together or separately. Both diseases are characterized by the development of autoantibodies targeting subcellular antigens, with an increased risk of cardiovascular morbidity [51]. The neutrophil function is dysregulated, leading to phagocytosis, oxidative bursts accompanied by the release of radical oxygen species (ROS), and the formation and spontaneous release of neutrophil extracellular traps (NETs). This process contributes to the autoimmune process [51].

Other conditions related to PE development, such as thrombocytopenic purpura (TTP) or COVID-19 infections, increase the risk of PE development during pregnancy by twofold [52,53].

To prevent clinical manifestation of PE, early anticoagulant treatment with low-molecular-weight heparin or low-dose aspirin in pregnant women of advanced maternal age with hypercoagulability is effective. It has been demonstrated that this management strategy can significantly improve the prognosis of both mothers and infants [54].

PE can be associated with autoimmune diseases and, in the context of clinically active inflammatory bowel disease, is associated with an increased risk of adverse pregnancy outcomes. In this regard, assessments of fecal calprotectin are necessary because this substance is associated with an increased risk of adverse pregnancy outcomes such as PE, eclampsia, abruption placentae or membrane rupture [24,52,55,56]. Hypoxic–ischemic encephalopathy in infants is associated with PE and is an important cause of morbidity and mortality globally [57]. Circulating calprotectin has shown to be a promising marker to evaluate the severity of PE and infant viability [58]. Calprotectin is linked to trophoblast abnormalities and can also be associated with the development and persistence of autoimmunity in APS, which may apply more broadly to human autoimmune diseases [59]. Autoimmune diseases can damage multiple organs, influencing quality of life and causing disability and death. Calprotectin secretion may exacerbate these diseases and indicate disease severity. Calprotectin was identified in the umbilical cord tissues and placenta of preeclamptic pregnant women [60].

An acute rise in ALP compared with healthy pregnancy may signify placental damage in severe preeclampsia, or may be associated with HELLP syndrome (22%); calprotectin levels have been shown to rise in parallel [61]. Calprotectin can induce a decrease in placental lactogen and an increase in inflammatory cytokines. Human placental lactogen is a precursor to vasoinhibin, which regulates blood vessel growth in the placenta, and its dysregulation plays a role in early-onset severe preeclampsia [62].

Calprotectin in placental tissues has been observed to play a role in fetal development and viability, as well as pregnancy complications such as PE and recurrent pregnancy loss [63].

Although Golubinskaya et al. found that preeclampsia was diagnosed in mothers with elevated calprotectin, they did not find a relationship between calprotectin and the severity of placental damage or neonatal viability [64].

In addition, preterm infants exposed to chorioamnionitis are at risk of neonatal morbidity and adverse outcomes. Alarmins S100A8, S100A9, and S100A12 are released by myeloid cells and neutrophils and have been associated with an inflammatory response. Researchers have studied the calprotectin expression in cord blood monocytes from preterm infants and term healthy infants, and found that S100A8 and S100A9 gene expression is significantly increased in preterm versus term infants [64].

Calprotectin is an innate immune effector with a role in the change in placental extracellular vesicles (EVs) in pregnant women with PE [65]. Extracellular vesicles (EVs) are lipid bilayer nanoparticles released from different cell types and are involved in cell-to-cell cross-talk [65]. They could be used to target NETs, scavenge ROS in fetuses suffering severe hypoxia, and reduce inflammatory factors in the case of PE and IUGR [46,66]. According to Braekke, a statistical significant correlation was not found in the preeclamptic group [36]. Calprotectin, a marker of inflammation, is elevated in the maternal but not in the fetal circulation in preeclampsia [36,67].

These findings are presented in Table 1.

To summarize, elevated calprotectin levels in preeclampsia suggest an underlying inflammatory process in the mother, potentially contributing to the development of this condition; however, more research is needed to clarify its role.

## 7. Current Issues and Future Perspectives

### 7.1. Neonatal Morbidity/Mortality

Hosono et al. (2006) enrolled 78 infants in their study. The one-year survival rates after 22, 23, and 24 weeks of gestation were between 40% and 61.1% (40.0%, 61.1%, and 50.0% in the 22-, 23-, and 24-week groups, respectively). The most frequent causes of death in the 23-week group were a lack of response to surfactants and severe respiratory distress, while a low Apgar score was associated with intraventricular hemorrhage (≥III). Sepsis was observed in infants born at 24 weeks of gestation. In addition, severe handicap rates were high in survivors born at 22-, 23-, and 24-week gestation, being 100% in the 22-week group, with severe neurological outcomes. This demonstrates that every possible effort should be made to extend gestation beyond 22 weeks [68].

We must also be aware of the possible disagreements between parents and professionals regarding the management of newborns, particularly in relation to the limits of viability and attitudes toward resuscitation. This also depends on the way information on morbidity/mortality risks and on “survival without disability” at 23 to 25 weeks is given or received. Parental information must be communicated in a way that subtly shapes the decisions that follow. Management plans should be made before birth, and more protocolized counseling, with supportive materials, is necessary [68,69,70].

In the case of congenital malformations, termination of pregnancy may be an option, even if the fetus is already viable. In Flanders, Belgium, there is a high degree of tolerance toward late termination of pregnancy, demanding legislative change regarding active life-ending in fetal and neonatal periods [71].

Most healthcare professionals would continue to administer intensive care to extremely preterm neonates or neonates with certain types of malformations, such as complete phocomelia. It is important to understand healthcare in the context of ethical dilemmas to support strategies and improve policies and, ultimately, the quality of neonatal intensive care [72].

#### Prolonging Gestation

An advanced form of therapy is recommended to safely prolong the duration of pregnancy in the case of preeclampsia. This type of management could reduce perinatal complications [5]. The aim of this expectant management is to lower blood pressure and prevent seizures. Nevertheless, premature birth may be inevitable and is associated with complications such as neonatal death, bronchopulmonary dysplasia, and intraventricular hemorrhage [73]. Various pre-existing maternal pathologies exacerbate the occurrence of PE, for example, chronic hypertension, antiphospholipid syndrome, systemic lupus erythematosus, thrombotic thrombocytopenic purpura, and diabetes mellitus. These pathologies create a permanent inflammatory state in which defective trophoblastic invasion with hypoxia and increased oxidative stress is maintained [9,10,73]. New therapeutic candidates have been proposed for the prevention or treatment of PE, including drugs that can affect the pathophysiology of the disease and placental or endothelial dysfunction, or both. Molecular targets such as oxidative stress, anti-angiogenic factors, and inflammatory pathways have also been proposed [74].

### 7.2. Antioxidant Therapy

By targeting oxidative stress and also reducing anti-angiogenic factors, we aim to prolong gestation in women with preeclampsia, alleviating endothelial dysfunction. Various therapeutic strategies are under investigation [73].

Gülmezoğlu et al. evaluated 56 women with severe preeclampsia between 24 and 32 weeks of gestation, comparing antioxidant combined therapy (200 mg allopurinol, 800 IU vitamin E, and 1000 mg vitamin C) with a placebo [75]. The trial showed a prolongation of pregnancy of 14 days in the antioxidant group compared with the control group [75]. Allopurinol was associated with a significant reduction in blood pressure [75,76]. This therapy is widely used as treatment for multiple diseases and has not been observed to lead to an increased risk of congenital anomalies [77,78]. It has also shown efficacy as an early intervention in preventing the development of cardiac dysfunction in adult offspring of hypoxic pregnancies [79]. Another study showed that allopurinol, when co-administrated with methyldopa, improves maternal and biochemical indicators of preeclampsia, reducing blood pressure [80].

### 7.3. Proton Pump Inhibitors

Inhibiting potassium–hydrogen ATPase through proton pump inhibitors showed no significant clinical benefit for treating PE. Cluver et al. used esomeprazole in their randomized trial of 120 women with PE, observing no significant effect on sFLT-1 and PlGF levels and no clinical improvement [81]. In addition, Neuman et al., in their trial of 50 pregnant women, also observed no benefit from using omeprazole in combination with standard treatment regarding prolonging pregnancy [82]. Two other trials using proton pump inhibitors, encompassing 177 women with preeclampsia, showed that they had no effect on HELLP syndrome or neonatal mortality. There are no ongoing trials to investigate their efficacy in the prevention of PE [83]. Another study has emphasized that PPI use in women at greatest risk of preterm preeclampsia may help prevent severe forms of disease [84].

### 7.4. Pravastatin

Pravastatin is a statin that has been investigated for its potential role in preeclampsia. It is believed to play a role in reducing sFLT-1 and increasing PlGF. In a randomized trial, 62 women with PE received pravastatin, but it failed to demonstrate effectiveness in prolonging pregnancy [85]. On the contrary, another study showed that prophylactic use of pravastatin reduced the risk of developing preeclampsia, also lowering the risk of preterm birth, IUGR, and NICU admission in neonates [75].

### 7.5. Metformin

Metformin is used for diabetes mellitus and also exhibits antioxidant effects [86]. It has been used in a phase II trial in South Africa on 180 women with PE. No significant changes were observed in sFLT-1 or PlGF levels, but its use was associated with a seven-day prolongation of pregnancy [87]. Metformin’s low cost and established safety during pregnancy make it a promising candidate for preterm preeclampsia treatment [66]. Cluver et al., in their study with 180 participants, also demonstrated that metformin can prolong gestation in women with preterm preeclampsia [87].

### 7.6. Plasma Apheresis to Reduce sFLT-1 Levels

Plasma apheresis is another strategy that is being tested to prolong gestation in preeclampsia, but the study has been interrupted due to safety issues [88]. In Haddad et al.’s (2018) phase II trial concerning early PE at <26 weeks gestation, LDL-apheresis did not result in a prolongation of pregnancy after one week. Two patients developed secondary uncontrolled hypertension, and thus the study was interrupted for safety reasons [88]. In another study, Winkler suggested specific removal of sFlt-1 via dextran sulfate cellulose (DSC)-apheresis as a cure for preterm PE, allowing for prolongation of pregnancy for 9 days under safe conditions [89].

### 7.7. Progesterone

Progesterone is another therapy that has been proposed for PE, which is characterized by low progesterone levels. The use of dydrogesterone before 20 weeks of gestation in women with high-risk factors for PE contributed to a statistically significant reduction in the frequency of this condition according to Tskhay et al. [90]. The same outcome was noted by Darzi et al. (2025) in their research on 60 women with a high risk of early PE using progesterone in addition to aspirin. This combination reduced the prevalence of preterm births [91].

### 7.8. Monoclonal Antibody

Eculizumab [74] or caplacizumab [52] may also have therapeutic potential.

### 7.9. Calprotectin Inhibitors

ABR-238901 is a calprotectin blocker which reduces ischemic injury and enhance angiogenesis, in an animal model study [92].

Quinoline-3-carboxamides have anti-inflammatory properties and are in phase II and III treatment under development for anti-inflammatory/autoimmune disease [93,94].

All these types of therapies are detailed in the table below (Table 2).

#### Strength and Limitations

Our review is important for its novelty, exploring the role of calprotectin in preeclampsia, its link to infant viability, and also its potential as targeted treatment. A limitation that should be mentioned here is that this is a narrative review, which explores the appropriate management of PE—including monitoring and therapeutic options to prolong gestation—based on variable study designs and different outcomes reported by researchers. Another limitation is that we failed to demonstrate clearly the importance of calprotectin in PE. In our results, we did not find sufficient evidence to reach firm conclusions regarding calprotectin use as a potential screening marker. However, we found sufficient evidence regarding appropriate monitoring tools in order to increase safety in PE and infant viability, which is highly relevant for clinical application.

## 8. Conclusions

In summary, early-stage preeclampsia remains a significant risk to maternal and neonatal health with significant impact on neonatal viability. Calprotectin could be a potential biomarker in preeclampsia as an important inflammation marker. But so far, calprotectin has failed to prove its role as a marker of fetal viability; thus, more studies are needed.

There is an urgent need for effective strategies to prolong pregnancy under safe conditions, which could substantially improve outcomes. The purpose of this intervention is to reduce neonatal complications, with a secondary goal of reducing healthcare costs. Calprotectin inhibitors are not yet recommended for safety reasons.

## Figures and Tables

**Table 1:** Calprotectin and preeclampsia.

References	Calprotectin Activity	Role in Placenta	Relevance in PE
[3]	Inflammation	Influence trophoblastic invasion	Role in PE, potential biomarker
[12,13,14,23]	Inflammation, calcium storage, metal scavenger, antimicrobial	Inflammation in placenta and decidua, leukocyte activation in placenta	Role in PE
[21,22]	Inflammation	Hypoxia/ischemia of placenta	The role of calprotectin in PE is controversial
[27]	Coagulopathy and increased fibrinolysis	Placental ischemia	Role in PE
[35]	Maternal leukocyte activation	Ischemia in placenta, inflammation	Role in PE
[36]	Inflammation in decidua, maternal and fetal plasma	Inflammation	Role in PE, potential biomarker
[37,38]	Pathogenesis of PE	Inflammation	Possible role but more research is needed
[39]	Inflammation, COVID-19 infection	Inflammation two times higher than normal	Role in PE
[40]	Calcium is a sensor for calprotectin	Placental ischemia	Role in PE
[14,41,42,43]	Increased level of plasma calprotectin	Placental insufficiency	Role in PE
[41,45]	Increased calprotectin in amniotic fluid, inflammation, infection	Placental ischemia	Role in PE
[45,46,47]	Neutrophil extracellular traps (NETs) and infections	Placental ischemia	Role in PE
[48,49,50]	NETs and non-infection stimuli, vasculopathy in autoimmune diseases	Placental ischemia	Role in PE
[51,52,53]	Antiphospholipid syndrome and vasculopathy	Placental ischemia	Role in PE
[24,52,55]	Fecal calprotectin associated with PE, placental abruption	Placental ischemia	Controversial
[58]	Plasma calprotectin associated with inflammation in mother and infant, role as biomarker	Placental ischemia Calprotectin associated with PE severity and infant viability	Role in PE
[60]	Calprotectin present in umbilical cord	Placental ischemia	Not known
[61]	Plasma calprotectin rise in parallel with severity of PE, biomarker	Placental ischemia calprotectin associated with PE severity and infant viability	Role in PE
[63]	Plasma calprotectin in mother and infants	Placental ischemia, fetal development and viability	Role in PE severity
[64]	Placental and plasma calprotectin, in mothers and infants	Severity of placental damage or neonatal viability	Role in PE
[65,66]	Calprotectin with effects on placental EVs	Placental ischemia and inflammation	Role in PE

**Table 2:** Types of therapies used to prolong gestation in the case of PE.

Type of Therapy	Effect	Reference	Conclusion
Antioxidant therapy/Allopurinol	Targets oxidative stress	[73]	Significant effect
	Reduces anti-angiogenic factors	[75]	Significant effect
	Safe for pregnancy Prevents the development of cardiac dysfunction in adult offspring of hypoxic pregnancies Improves the maternal and biochemical indicators of preeclampsia, reducing blood pressure	[77,78,79,80]	Further studies needed Further studies needed
Proton pump inhibitors/esomeprazole	Act on sFLT-1 and PlGF level	[73]	No significant effect
Proton pump inhibitors omeprazole	Act on sFLT-1 and PlGF levels	[73]	No significant effect
Proton pump inhibitors	Prevents severe forms of the disease	[83,84]	Trials ongoing
Pravastatin	Reduces sFLT-1 and increases PlGF	[73,85]	Failed to demonstrate an effect
Pravastatin	Prophylactic use	[86]	Significant effect
Metformin	Antioxidant effect sFLT-1 or PlGF levels	[87,88]	Further research needed
Plasmapheresis	Reduces sFLT-1 levels	[82,83]	Study interrupted for safety reasons
Progesterone	Increases progesterone levels, which are low in PE	[90,91]	Statistically significant
Monoclonal antibody Eculizumab, caplacizumab	Scavenging	[52,74]	Promising effect
Quinoline-3-carboxamides	Anti-inflammatory effect	[93,94]	Promising effect
ABR-238901	reduces ischemic injury and enhance angiogenesis	[92]	Promising effect

## Data Availability

Not applicable.

## Abbreviations

The following abbreviations are used in this manuscript:

PE	preeclampsia
IUGR	intrauterine growth restriction
ISUOG	International Society of Ultrasound in Obstetrics and Gynecology
FGR	fetal growth restriction
TAS	systolic blood pressure
TAD	diastolic blood pressure
CTG	cardiotocography
PAPPA	plasma protein-A
sFlt-1	soluble fms-like tyrosine kinase-1
PlGF	placental growth factor
VEGF	vascular endothelial growth factor receptor-1
SMFM	Society for Maternal–Fetal Medicine
HELLP syndrome	hemolysis, elevated liver enzymes, low platelets
NICA	neonatal intensive care
MVM	maternal vascular malperfusion
FVM	fetal vascular malperfusion
ACA	Acute Chorioamnionitis
VUE	Villitis of Unknown Etiology
IL-6	interleukin 6
NETs	neutrophil extracellular traps
SLE	systemic lupus erythematosus
RA	rheumatoid arthritis
APS	antiphospholipid syndrome
aPLs	antiphospholipid antibodies
TTP	thrombotic thrombocytopenic purpura
ROS	radical oxygen species
EVs	extracellular vesicles

## References

[1] American College of Obstetricians and Gynecologists (2006). ACOG practice bulletin: Clinical management guidelines for obstetrician-gynecologists number 76, October 2006: Postpartum hemorrhage. Obstet. Gynecol..

[2] Williamson R. (2019). Pre-Clinical Characterisation of Mitochondrial Antioxidants as Novel Therapeutics for Pre-Eclampsia. Ph.D. Thesis.

[3] Black K.D., Horowitz J.A. (2018). Inflammatory markers and preeclampsia: A systematic review. Nurs. Res..

[4] Roten L.T. (2009). Genetic Predisposition for Development of Preeclampsia: Candidate Gene Studies in the HUNT (Nord-Trøndelag Health Study) Population. Ph.D.Thesis.

[5] American College of Obstetricians and Gynecologists (2019). ACOG Practice Bulletin No. 204: Fetal growth restriction. Obstet. Gynecol..

[6] Lees C.C., Stampalija T., Baschat A., da Silva Costa F., Ferrazzi E., Figueras F., Unterscheider J. (2020). ISUOG Practice Guidelines: Diagnosis and management of small-for-gestational-age fetus and fetal growth restriction. Ultrasound Obstet. Gynecol..

[7] Rodriguez-Sibaja M.J., Lopez-Diaz A.J., Valdespino-Vazquez M.Y., Acevedo-Gallegos S., Amaya-Guel Y., Camarena-Cabrera D.M., Lumbreras-Marquez M.I. (2024). Placental pathology lesions: International Society for Ultrasound in Obstetrics and Gynecology vs Society for Maternal-Fetal Medicine fetal growth restriction definitions. Am. J. Obstet. Gynecol. MFM.

[8] Kvehaugen A.S., Dechend R., Ramstad H.B., Troisi R., Fugelseth D., Staff A.C. (2011). Endothelial function and circulating biomarkers are disturbed in women and children after preeclampsia. Hypertension.

[9] Mitranovici M.I., Chiorean D.M., Moraru R., Moraru L., Caravia L., Tiron A.T., Cotoi O.S. (2024). Understanding the pathophysiology of preeclampsia: Exploring the role of antiphospholipid antibodies and future directions. J. Clin. Med..

[10] Buicu C.F., Mitranovici M.I., Voidazan S., Craina M. (2025). Metoprolol Use in Hypertensive Pregnancy Disorder—A Single-center Study. J. Cardiovasc. Emergencies.

[11] Wright D., Poon L.C., Rolnik D.L., Syngelaki A., Delgado J.L., Vojtassakova D., Nicolaides K.H. (2017). Aspirin for Evidence-Based Preeclampsia Prevention trial: Influence of compliance on beneficial effect of aspirin in prevention of preterm preeclampsia. Am. J. Obstet. Gynecol..

[12] Holthe M.R., Staff A.C., Berge L.N., Fagerhol M.K., Lyberg T. (2005). Calprotectin plasma level is elevated in preeclampsia. Acta Obstet. Gynecol. Scand..

[13] Singh P., Ali S.A. (2022). Multifunctional role of S100 protein family in the immune system: An update. Cells.

[14] Pergialiotis V., Prodromidou A., Pappa E., Vlachos G.D., Perrea D.N., Papantoniou N. (2016). An evaluation of calprotectin as serum marker of preeclampsia: A systematic review of observational studies. Inflamm. Res..

[15] Bouter A.R., Duvekot J.J. (2020). Evaluation of the clinical impact of the revised ISSHP and ACOG definitions on preeclampsia. Pregnancy Hypertens.

[16] Dymara-Konopka W., Laskowska M., Oleszczuk J. (2018). Preeclampsia-current management and future approach. Curr. Pharm. Biotechnol..

[17] Stampalija T., Lees C., Ghi T., Cornette J., Gyselaers W., Ferrazzi E., Mousa T., Spaanderman M., Thilaganathan B., Valensise H. (2025). ISUOG Consensus Statement on maternal hemodynamic assessment in hypertensive disorders of pregnancy and fetal growth restriction. Ultrasound Obstet. Gynecol..

[18] Alnaes-Katjavivi P., Roald B., Staff A.C. (2020). Uteroplacental acute atherosis in preeclamptic pregnancies: Rates and clinical outcomes differ by tissue collection methods. Pregnancy Hypertens.

[19] Chiorean D.M., Cobankent Aytekin E., Mitranovici M.I., Turdean S.G., Moharer M.S., Cotoi O.S., Toru H.S. (2024). Human placenta and evolving insights into pathological changes of preeclampsia: A comprehensive review of the last decade. Fetal Pediatr. Pathol..

[20] Schiopu A., Cotoi O.S. (2013). S100A8 and S100A9: DAMPs at the crossroads between innate immunity, traditional risk factors, and cardiovascular disease. Mediat. Inflamm..

[21] Than N.G., Romero R., Goodman M., Weckle A., Xing J., Dong Z., Xu Y., Tarquini F., Szilagyi A., Gal P. (2009). A primate subfamily of galectins expressed at the maternal–fetal interface that promote immune cell death. Proc. Natl. Acad. Sci. USA.

[22] Vince G.S., Starkey P.M., Austgulen R., Kwiatkowski D., Redman C.W.G. (1995). Interleukin-6, turnour necrosis factor and soluble turnour necrosis factor receptors in women with pre-eclampsia. BJOG Int. J. Obstet. Gynaecol..

[23] Berkane N., Liere P., Oudinet J.P., Hertig A., Lefèvre G., Pluchino N., Schumacher M., Chabbert-Buffet N. (2017). From pregnancy to preeclampsia: A key role for estrogens. Endocr. Rev..

[24] Sibai B.M., Publications Committee, Society for Maternal-Fetal Medicine (2011). Evaluation and management of severe preeclampsia before 34 weeks’ gestation. Am. J. Obstet. Gynecol..

[25] Redline R.W., Ravishankar S., Bagby C.M., Saab S.T., Zarei S. (2021). Four major patterns of placental injury: A stepwise guide for understanding and implementing the 2016 Amsterdam consensus. Mod. Pathol..

[26] Stepan H., Hund M., Andraczek T. (2020). Combining biomarkers to predict pregnancy complications and redefine preeclampsia: The angiogenic-placental syndrome. Hypertension.

[27] Godtfredsen A.C., Sidelmann J.J., Dolleris B.B., Jørgensen J.S., Johansen E.K.J., Pedersen M.F.B., Palarasah Y., Gram J.B. (2022). Fibrinolytic changes in women with preeclampsia. Clin. Appl. Thromb. Hemost..

[28] Crocker I.P., Cooper S., Ong S.C., Baker P.N. (2003). Differences in apoptotic susceptibility of cytotrophoblasts and syncytiotrophoblasts in normal pregnancy to those complicated with preeclampsia and intrauterine growth restriction. Am. J. Pathol..

[29] Chiang Y.T., Seow K.M., Chen K.H. (2024). The pathophysiological, genetic, and hormonal changes in preeclampsia: A systematic review of the molecular mechanisms. Int. J. Mol. Sci..

[30] Michita R.T., Kaminski V.D.L., Chies J.A.B. (2018). Genetic variants in preeclampsia: Lessons from studies in Latin-American populations. Front. Physiol..

[31] Irimia T., Pușcașiu L., Mitranovici M.-I., Crișan A., Budianu M.A., Bănescu C., Chiorean D.M., Niculescu R., Sabău A.-H., Cocuz I.-G. (2022). Oxidative-stress related gene polymorphism in endometriosis-associated infertility. Medicina.

[32] Bombrys A.E., Barton J.R., Nowacki E.A., Habli M., Pinder L., How H., Sibai B.M. (2008). Expectant management of severe preeclampsia at less than 27 weeks’ gestation: Maternal and perinatal outcomes according to gestational age by weeks at onset of expectant management. Am. J. Obstet. Gynecol..

[33] Witlin A.G., Saade G.R., Mattar F., Sibai B.M. (2000). Predictors of neonatal outcome in women with severe preeclampsia or eclampsia between 24 and 33 weeks’ gestation. Am. J. Obstet. Gynecol..

[34] Shear R.M., Rinfret D., Leduc L. (2005). Should we offer expectant management in cases of severe preterm preeclampsia with fetal growth restriction?. Am. J. Obstet. Gynecol..

[35] Holthe M.R., Lyberg T., Staff A.C., Berge L.N. (2005). Leukocyte-platelet interaction in pregnancies complicated with preeclampsia. Platelets.

[36] Braekke K., Holthe M.R., Harsem N.K., Fagerhol M.K., Staff A.C. (2005). Calprotectin, a marker of inflammation, is elevated in the maternal but not in the fetal circulation in preeclampsia. Am. J. Obstet. Gynecol..

[37] Rezniczek G.A., Förster C., Hilal Z., Westhoff T., Tempfer C.B. (2019). Calprotectin in pregnancy and pregnancy-associated diseases: A systematic review and prospective cohort study. Arch. Gynecol. Obstet..

[38] Katiyar H., Yadav S., Singh S., Mishra A.K., Pradhan M., Lingaiah R., Goel A. (2024). Evaluation of Serum Calprotectin as an Alternative Diagnostic Marker for Intrahepatic Cholestasis of Pregnancy. J. Clin. Med..

[39] Michaud A., Ng-Pellegrino A., Birk R., Stawicki S.P. (2022). The 2022 St. Luke’s University health network annual research symposium: Event highlights and scientific abstracts. Int. J. Acad. Med..

[40] Montecinos L., Eskew J.D., Smith A. (2019). What is next in this “age” of heme-driven pathology and protection by hemopexin? An update and links with iron. Pharmaceuticals.

[41] Goldmann O., Medina E. (2013). The expanding world of extracellular traps: Not only neutrophils but much more. Front. Immunol..

[42] Jiménez-Cortegana C., Salamanca E., Palazón-Carrión N., Sánchez-Jiménez F., Pérez-Pérez A., Vilariño-García T., Fuentes S., Martín S., Jiménez M., Galván R. (2023). Circulating myeloid-derived suppressor cells may be a useful biomarker in the follow-up of unvaccinated COVID-19 patients after hospitalization. Front. Immunol..

[43] Marković S., Popović T., Jelušić A., Iličić R., Stanković S. Genetic Insight into the Isolates Causing Blackleg Disease on Potato. Proceedings of the 6th Congress of the Serbian Genetic Society.

[44] Aberšek N., Tsiartas P., Jonsson D., Grankvist A., Barman M., Hallingström M., Jacobsson B. (2022). Calprotectin levels in amniotic fluid in relation to intra-amniotic inflammation and infection in women with preterm labor with intact membranes: A retrospective cohort study. Eur. J. Obstet. Gynecol. Reprod. Biol..

[45] Moodley M., Moodley J., Naicker T. (2020). Neutrophil extracellular traps: The synergy source in the placentae of HIV infected women with pre-eclampsia. Pregnancy Hypertens.

[46] Zhou Y., Xu L., Jin P., Li N., Chen X., Yang A., Qi H. (2024). NET-targeted nanoparticles for antithrombotic therapy in pregnancy. Iscience.

[47] Kaplan M.J., Radic M. (2012). Neutrophil extracellular traps: Double-Edged swords of innate immunity. J. Immunol..

[48] Bonaventura A., Liberale L., Carbone F., Vecchié A., Diaz-Cañestro C., Camici G.G., Montecucco F., Dallegri F. (2018). The pathophysiological role of neutrophil extracellular traps in inflammatory diseases. Thromb. Haemost..

[49] Brinkmann V. (2018). Neutrophil extracellular traps in the second decade. J. Innate Immun..

[50] Hoppenbrouwers T., Autar A.S.A., Sultan A.R., Abraham T.E., van Cappellen W.A., Houtsmuller A.B., van Wamel W.J.B., van Beusekom H.M.M., van Neck J.W., de Maat M.P.M. (2017). In vitro induction of NETosis: Comprehensive live imaging comparison and systematic review. PLoS ONE.

[51] Wirestam L., Arve S., Linge P., Bengtsson A.A. (2019). Neutrophils—Important communicators in systemic lupus erythematosus and antiphospholipid syndrome. Front. Immunol..

[52] Mitranovici M.I., Pușcașiu L., Oală I.E., Petre I., Craina M.L., Mager A.R., Vasile K., Chiorean D.M., Sabău A.-H., Turdean S.G. (2022). A race against the clock: A case report and literature review concerning the importance of ADAMTS13 testing in diagnosis and management of thrombotic thrombocytopenic purpura during pregnancy. Diagnostics.

[53] Mitranovici M.I., Chiorean D.M., Oală I.E., Petre I., Cotoi O.S. (2022). Evaluation of the Obstetric Patient: Pregnancy Outcomes during COVID-19 Pandemic—A Single-Center Retrospective Study in Romania. Reports.

[54] Deng Y., She L., Li X., Lai W., Yu L., Zhang W., Peng M. (2022). Monitoring hypertensive disorders in pregnancy to prevent preeclampsia in pregnant women of advanced maternal age: Trial mimicking with retrospective data. Open Med..

[55] Prentice R.E., Flanagan E.K., Wright E.K., Dolinger M.T., Gottlieb Z., Ross A.L., Bell S.J. (2025). Active inflammatory bowel disease on intestinal ultrasound during pregnancy is associated with an increased risk of adverse pregnancy and neonatal outcomes independent of clinical and biochemical disease activity. Gastroenterology.

[56] Campbell C., Kandalgaonkar M.R., Golonka R.M., Yeoh B.S., Vijay-Kumar M., Saha P. (2023). Crosstalk between gut microbiota and host immunity: Impact on inflammation and immunotherapy. Biomedicines.

[57] Yip P.K., Bremang M., Pike I., Ponnusamy V., Michael-Titus A.T., Shah D.K. (2023). Newborns with favourable outcomes after perinatal asphyxia have upregulated glucose metabolism-related proteins in plasma. Biomolecules.

[58] Mahroum N., Elsalti A., Alwani A., Seida I., Alrais M., Seida R., Esirgun S.N., Abali T., Kiyak Z., Zoubi M. (2022). The mosaic of autoimmunity–Finally discussing in person. The 13th international congress on autoimmunity 2022 (AUTO13) Athens. Autoimmun. Rev..

[59] Ruff W.E., Dehner C., Kim W.J., Pagovich O., Aguiar C.L., Yu A.T., Roth A.S., Vieira S.M., Kriegel C., Adeniyi O. (2019). Pathogenic autoreactive T and B cells cross-react with mimotopes expressed by a common human gut commensal to trigger autoimmunity. Cell Host Microbe.

[60] Ren R., Jiang J., Li X., Zhang G. (2024). Research progress of autoimmune diseases based on induced pluripotent stem cells. Front. Immunol..

[61] Morton A. (2024). Investigating gastrointestinal disorders in pregnancy. Obstet. Med..

[62] Singh G., Prentice R., Langsford D., Christensen B., Garg M. (2021). Altered bowel habit and rectal bleeding in pregnancy: The importance of recognising undiagnosed inflammatory bowel disease. Intern. Med. J..

[63] Li L., Baek K.H. (2025). Exploring Potential Biomarkers in Recurrent Pregnancy Loss: A Literature Review of Omics Studies to Molecular Mechanisms. Int. J. Mol. Sci..

[64] Golubinskaya V., Puttonen H., Fyhr I.-M., Rydbeck H., Hellström A., Jacobsson B., Nilsson H., Mallard C., Sävman K. (2020). Expression of S100A alarmins in cord blood monocytes is highly associated with chorioamnionitis and fetal inflammation in preterm infants. Front. Immunol..

[65] Al-Jipouri A., Eritja À., Bozic M. (2023). Unraveling the Multifaceted Roles of Extracellular Vesicles: Insights into Biology, Pharmacology, and Pharmaceutical Applications for Drug Delivery. Int. J. Mol. Sci..

[66] Kaluarachchi A., Peiris G.R.M.U.G., Ranaweera A.K.P., Rishard M.R.M. (2018). Hyperechoic amniotic fluid in a term pregnancy. J. Fam. Med. Prim. Care.

[67] Sugulle M., Kvehaugen A.S., Brække K., Harsem N.K., Staff A.C. (2011). Plasma calprotectin as inflammation marker in pregnancies complicated by diabetes mellitus and superimposed preeclampsia. Pregnancy Hypertens.

[68] Hosono S., Ohno T., Kimoto H., Shimizu M., Harada K. (2006). Morbidity and mortality of infants born at the threshold of viability: Ten years’ experience in a single neonatal intensive care unit, 1991–2000. Pediatr. Int..

[69] Papadimitriou V., Tosello B., Pfister R. (2019). Effect of written outcome information on attitude of perinatal healthcare professionals at the limit of viability: A randomized study. BMC Med. Ethics.

[70] Lui K., Bajuk B., Foster K., Gaston A., Kent A., Sinn J., Spence K., Fischer W., Henderson-Smart D. (2006). Perinatal care at the borderlines of viability: A consensus statement based on a NSW and ACT consensus workshop. Med. J. Aust..

[71] Roets E., Dierickx S., Deliens L., Chambaere K., Dombrecht L., Roelens K., Beernaert K. (2021). Healthcare professionals’ attitudes towards termination of pregnancy at viable stage. Acta Obstet. Gynecol. Scand..

[72] Dagla M., Petousi V., Poulios A. (2021). Neonatal end-of-life decision making: The possible behavior of Greek physicians, midwives, and nurses in clinical scenarios. Int. J. Environ. Res. Public Health.

[73] Provinciatto H., Araujo Júnior E., Granese R. (2025). Therapeutic strategies to prolong gestation in preterm preeclampsia. J. Obstet. Gynaecol..

[74] Tong S., Tu’uhevaha J., Hastie R., Brownfoot F., Cluver C., Hannan N. (2022). Pravastatin, proton-pump inhibitors, metformin, micronutrients, and biologics: New horizons for the prevention or treatment of preeclampsia. Am. J. Obstet. Gynecol..

[75] Gülmezoǧlu A.M., Hofmeyr G.J., Oosthuisen M.M.J. (1997). Antioxidants in the treatment of severe pre-eclampsis an explanatory randomised controlled trial. BJOG: Int. J. Obstet. Gynaecol..

[76] Madero M., Castellanos F.E.R., Jalal D., Villalobos-Martín M., Salazar J., Vazquez-Rangel A., Johnson R.J., Sanchez-Lozada L.G. (2015). A pilot study on the impact of a low fructose diet and allopurinol on clinic blood pressure among overweight and prehypertensive subjects: A randomized placebo controlled trial. J. Am. Soc. Hypertens.

[77] Crouwel F., Simsek M., de Boer M.A., van Asseldonk D.P., Bhalla A., Weusthuis A.L.M., Gilissen L.P.L., Verburg R.J., Mares W.G.N., Jharap B. (2024). Multicentre study and systematic review: Allopurinol exposure during pregnancy. Aliment. Pharmacol. Ther..

[78] Simsek M., Opperman R.C., Mulder C.J., Lambalk C.B., de Boer N.K. (2018). The teratogenicity of allopurinol: A comprehensive review of animal and human studies. Reprod. Toxicol..

[79] Niu Y., Kane A.D., Lusby C.M., Allison B.J., Chua Y.Y., Kaandorp J.J., Nevin-Dolan R., Ashmore T.J., Blackmore H.L., Derks J.B. (2018). Maternal allopurinol prevents cardiac dysfunction in adult male offspring programmed by chronic hypoxia during pregnancy. Hypertension.

[80] Ahmed G.S., Al-Sabbagh M.S. (2010). The Possible beneficial effects of Antioxidant drugs (Vitamin C and E) and Allopurinol in the management of Pre-eclamptic patients treated with Methyldopa. J. Fac. Med. Baghdad.

[81] Cluver C.A., Hannan N.J., van Papendorp E., Hiscock R., Beard S., Mol B.W., Theron G.B., Hall D.R., Decloedt E.H., Stander M. (2018). Esomeprazole to treat women with preterm preeclampsia: A randomized placebo controlled trial. Am. J. Obstet. Gynecol..

[82] Neuman R.I., Baars M.D., Saleh L., Broekhuizen M., Nieboer D., Cornette J., Schoenmakers S., Verhoeven M., Koch B.C., Russcher H. (2022). Omeprazole administration in preterm preeclampsia: A randomized controlled trial to study its effect on sFlt-1 (soluble Fms-like tyrosine kinase-1), PlGF (placental growth factor), and ET-1 (endothelin-1). Hypertension.

[83] Mills K., McDougall A.R., Tan A., Makama M., Nguyen P.-Y., Armari E., Bradfield Z., Hastie R., Ammerdorffer A., Gülmezoglu A.M. (2024). The effects of proton pump inhibitors during pregnancy on treatment of preeclampsia and related outcomes: A systematic review and meta-analysis. Am. J. Obstet. Gynecol. MFM.

[84] Hastie R., Bergman L., Cluver C.A., Wikman A., Hannan N.J., Walker S.P., Wikström A.K., Tong S., Hesselman S. (2019). Proton pump inhibitors and preeclampsia risk among 157 720 women: A Swedish population register–based cohort study. Hypertension.

[85] Ahmed A., Williams D.J., Cheed V., Middleton L.J., Ahmad S., Wang K., Vince A.T., Hewett P., Spencer K., Khan K.S. (2020). Pravastatin for early-onset pre-eclampsia: A randomised, blinded, placebo-controlled trial. BJOG: Int. J. Obstet. Gynaecol..

[86] Mészáros B., Veres D.S., Nagyistók L., Somogyi A., Rosta K., Herold Z., Kukor Z., Valent S. (2023). Pravastatin in preeclampsia: A meta-analysis and systematic review. Front. Med..

[87] Cluver C.A., Hiscock R., Decloedt E.H., Hall D.R., Schell S., Mol B.W., Brownfoot F., Kaitu’u-Lino T.J., Walker S.P., Tong S. (2021). Use of metformin to prolong gestation in preterm pre-eclampsia: Randomised, double blind, placecontrolled trial. BMJ.

[88] Haddad B., Lefèvre G., Rousseau A., Robert T., Saheb S., Rafat C., Bornes M., Petit-Hoang C., Richard F., Lecarpentier E. (2018). LDL-apheresis to decrease sFlt-1 during early severe preeclampsia: Report of two cases from a discontinued phase II trial. Eur. J. Obstet. Gynecol. Reprod. Biol..

[89] Winkler K., Contini C., König B., Krumrey B., Pütz G., Zschiedrich S., Pecks U., Stavropoulou D., Prömpeler H., Kunze M. (2018). Treatment of very preterm preeclampsia via heparin-mediated extracorporeal LDL-precipitation (HELP) apheresis: The Freiburg preeclampsia HELP-Apheresis study. Pregnancy Hypertens.

[90] Tskhay V., Schindler A., Shestakova M., Klimova O., Narkevich A. (2020). The role of progestogen supplementation (dydrogesterone) in the prevention of preeclampsia. Gynecol. Endocrinol..

[91] Darzi S., Arabian S., Kia P.M., Pordanjani S.R., Saffarieh E. (2025). Vaginal Progesterone Effects on Ultrasound Indices, Fetal Outcomes, and Preeclampsia in High-risk Pregnancy: Vaginal Progesterone in High-risk Pregnancy: Ultrasound Indices and Outcomes. Galen. Med. J..

[92] Mares R.G., Suica V.I., Uyy E., Boteanu R.M., Ivan L., Cocuz I.G., Sabau A.H., Yadav V., Szabo I.A., Cotoi O.S. (2024). Short-term S100A8/A9 Blockade Promotes Cardiac Neovascularization after Myocardial Infarction. J. Cardiovasc. Transl. Res..

[93] Björk P., Björk A., Vogl T., Stenström M., Liberg D., Olsson A., Roth J., Ivars F., Leanderson T. (2009). Identification of human S100A9 as a novel target for treatment of autoimmune disease via binding to quinoline-3-carboxamides. PLoS Biol..

[94] Bengtsson A.A., Sturfelt G., Lood C., Rönnblom L., Van Vollenhoven R.F., Axelsson B., Sparre B., Tuvesson H., Öhman M.W., Leanderson T. (2012). Pharmacokinetics, tolerability, and preliminary efficacy of paquinimod (ABR-215757), a new quinoline-3-carboxamide derivative: Studies in lupus-prone mice and a multicenter, randomized, double-blind, placebo-controlled, repeat-dose, dose-ranging study in patients with systemic lupus erythematosus. Arthritis Rheum..

